# Comparison of Pteridine Normalization Methods in Urine for Detection of Bladder Cancer

**DOI:** 10.3390/diagnostics10090612

**Published:** 2020-08-20

**Authors:** Piotr Kośliński, Robert Pluskota, Katarzyna Mądra-Gackowska, Marcin Gackowski, Michał J. Markuszewski, Kornelia Kędziora-Kornatowska, Marcin Koba

**Affiliations:** 1 Department of Toxicology and Bromatology, Collegium Medicum in Bydgoszcz, Nicolaus Copernicus University in Toruń, dr. A. Jurasza 2, 85-089 Bydgoszcz, Poland; robert.pluskota@cm.umk.pl (R.P.); marcin.gackowski@cm.umk.pl (M.G.); kobamar@cm.umk.pl (M.K.); 2 Department of Geriatrics, Collegium Medicum in Bydgoszcz, Nicolaus Copernicus University in Toruń, Poland, dr. A. Jurasza 2, 85-089 Bydgoszcz, Poland; katarzyna.madra@cm.umk.pl (K.M.-G.); kornelia.kornatowska@cm.umk.pl (K.K.-K.); 3 Department of Biopharmaceutics and Pharmacodynamics, Medical University of Gdańsk, al. Gen. J. Hallera 107, 80-416 Gdańsk, Poland; michal.markuszewski@gumed.edu.pl

**Keywords:** pterin, genitourinary cancer, creatinine, specific gravity

## Abstract

Pterin compounds belong to the group of biomarkers for which an increase in interest has been observed in recent years. Available literature data point to this group of compounds as potential biomarkers for cancer detection, although the biochemical justification for this claim is not yet fully understood. The aim of this study was to evaluate the usefulness of pterin compounds in the diagnosis of bladder cancer, with particular emphasis on the role of creatinine and the specific gravity of urine as factors for normalizing the concentration of pterin compounds in urine. The standardization of the concentration of pterin compounds to urine specific gravity allows the building of better classification models for screening patients with potential cancer of the bladder. Of the compounds that make up the pterin profile, isoxanthopterin appears to be a compound that can potentially be described as a biomarker of bladder cancer.

## 1. Introduction

The aim of the neoplastic diagnosis of the bladder is to recognize and assess the stage of the disease. In order to treat bladder tumors more effectively, the prevention and effective early detection of the disease are necessary. Currently, the search for new biomarkers or marker panels to improve diagnosis is progressing rapidly. In addition, clinically useful tools for monitoring therapy as well as detecting relapse can help to significantly improve outcomes and predict disease progression. The complexity and interrelationships of the metabolic pathways involved in cancer biology necessitate specific and sensitive markers for capturing clinically relevant changes in multiple metabolic pathways of carcinogenesis and tumor behavior. The rapidly changing consignment of metabolomics research represents a promising approach to developing effective cancer diagnosis and treatment strategies by monitoring fluctuations in the levels of certain metabolites in cells, tissues and biological fluids and establishing clinically useful diagnostic computational models [1].

Recently, an increase in the importance of tumor biomarkers in both diagnostic and therapeutic process has been observed. Cancer biomarkers are chemical compounds whose concentrations change during the neoplastic process. Biomarkers are compounds that provide diagnostic and prognostic information about biological processes that are changing in response to an ongoing disease process. Understanding what features of a biomarker make it suitable for determining and explaining the organism’s response to the disease process is crucial in the diagnostics [2,3,4].

Cancer biomarkers depending on their potential application can be divided into several categories: diagnostic biomarkers, prognostic biomarkers and predictive biomarkers [5].

The high cost and limitations such as the low sensitivity and specificity of current screening and diagnostic tests promote the search for alternative biomarkers for bladder cancer. These biomarkers should allow for early diagnosis and demonstrate high sensitivity and specificity, and the methods used for their determination should be affordable and widely applicable. The literature describes many tumor markers as potentially useful in the diagnosis and monitoring of patients with bladder cancer. They differ in their sensitivity, specificity and the methods used to determine them. However, only a small number of the markers tested are used in routine diagnosis [1]. The most common diagnostic tests performed for bladder cancer include urine cytology [6,7], hematuria detection [8,9,10], bladder tumor antigen detection (BTA) [7,11,12,13], the UroVysion test [14,15] and nuclear matrix protein 22 detection [16,17,18]. The mentioned tests differ significantly in sensitivity and diagnostic specificity for bladder cancer. The sensitivity of the cytological diagnosis ranges between 16 and 90% [6,7]. Despite the high specificity of hematuria detection (65–99%), only 0.7–1.3% of patients with hematuria suffer from bladder cancer [8,9,10]. The sensitivity of tests used to detect BTA ranges from 13 to 55% for low-grade (G1) tumors, 36 to 67% for G2 tumors and 63 to 90% for G3 tumors. In patients with non-neoplastic diseases of the urinary system, a significant decrease in the specificity of the test can be observed [7,11,12,13]. Many researchers have shown that the UroVysion test has a much higher sensitivity than the cytology of the urine sediment in all stages of the clinical advancement of bladder tumors. According to the literature data, this test is also characterized by a high specificity (about 95%), comparable to the specificity of the cytological testing of the urine sediment (about 94%) [14,15].

Pteridines constitute a large and structurally varied group of natural compounds involved in the biosynthetic pathways of cofactors and vitamins. Their derivatives are designated by the terms “pterins”. Pterins are a complex group of biological compounds thath have a characteristic ring structure. Pterins belong to a family of nitrogen heterocyclic compounds with a 2-amino-4-hydroxypteridine structure in their moieties. Various pterin derivatives have been extensively isolated from almost all kinds of living organisms. In nature, pterins occur in two major classes: so-called conjugated pterins, which are characterized by relatively complex chains (folic acid and derivatives), and unconjugated pterins possessing short chains. Besides neopterin or BH4, which has been recognized as the most important unconjugated pterin, there are other important pteridines that have been derived from the BH4 metabolism pathway. These compounds include xanthopterin, isoxanthopterin, 6,7-dimethylpterin, 6-biopterin, 6-xydroxymethylpterin, pterin and pterin-6-carboxylic acid [19,20]. Available literature data indicate this group of compounds as potential biomarkers for cancer detection, although the biochemical justification for this claim is not yet fully understood [19,20,21]. Despite many promising studies, the clinical utility of pterin compounds is still under discussion [22]. To date, no unequivocal reasons for the increased concentration of pterin compounds in cancer patients have been identified. The authors list three possible reasons for the change in the metabolic profile of pterins. These include (i) an increase in the level of biosynthesis, which leads to an increase in the metabolism of pterin compounds, (ii) an increase in catabolism without a significant increase in biosynthesis, or (iii) an increase in urinary excretion without a simultaneous increase in biosynthesis. However, observations indicating an increase in the blood levels of tetrahydrobiopterin in patients with various types of cancer suggests that the observed differences between healthy and sick individuals may result from an acceleration of pterin synthesis, which may lead to their increased metabolism. On the other hand, cell culture experiments indicate an increase in the catabolism of folic acid (or one of its derivatives) in neoplastic cells, which may also contribute to the higher excretion of some pterins in the urine [19]. This may also explain folate deficiency, which is observed in many types of neoplastic diseases. The use of creatinine as an indicator for normalizing the concentration of urine in relation to pterin compounds excreted in urine is also a subject of discussion [23,24].

The biological material used for the determination of various biomarkers is usually blood or urine. Due to the non-invasive nature of the collection and the possibility of obtaining a large amount of material, urine seems to be more widely used in screening diagnostics. A potential limitation in the use of urinary biomarkers is the need to take into account the relationship between their concentration and the patient’s hydration level and the time since the last urination. While creatine-based corrections are used in many subjects, the usefulness of this indicator can often be discounted [25,26].

Creatinine, which is a byproduct of muscle metabolism, is constantly removed from the blood by the kidneys, which became the basis for its use as a measure of the dilution of urine. Changes in the creatine concentration and thus its excretion were, however, associated with variables such as age, race and gender [27,28]; physical activity and muscle mass [29]; diet [30]; normal physiological functions, including menstrual cycles [31]; and pathological conditions [32,33]. These observations question the universality of creatinine as a factor for normalizing urine assays.

The second factor for normalizing the concentration of biomarkers in urine is specific gravity (USG). USG is commonly used as an alternative factor for the dilution of the urine instead of creatinine [34,35]. However, specific gravity measurement currently has limited utility in clinical settings, probably due to the lack of appropriate performance assessments [36,37]. Although the USG is subject to similar changes as creatinine [38], the benefits of its use are mainly due to the ease of correction using routine clinical urine tests. Furthermore, the USG can withstand many freezing/thawing cycles and long-term storage at −20 °C or lower [39].

The aim of this study was to assess the usefulness of pterin compounds in the diagnosis of bladder cancer with particular emphasis on the role of creatinine and the specific gravity of urine as factors for normalizing the concentration of pterin compounds in urine.

## 2. Results and Discussion

### 2.1. Basic Analyses

#### 2.1.1. Characteristics of Population

The group of patients with bladder cancer consisted of 31 people (14 women and 17 men). The average age for this study group was 64 years. The control group consisted of 32 people (11 men and 21 women). The average age for this study group was 75 years. All the variables examined on a quantitative scale had a non-normal distribution.

#### 2.1.2. Comparative Analysis of Concentrations of Pterin Compounds in the Studied Groups

The methods for standardizing the concentration of pterin compounds included two methods:

(i) Standardization using urine creatinine;
(ii) Standardization using urine specific gravity.

A comparison of the combined results from all the study groups obtained in accordance with the above standardization methods showed that the concentration of these pterin compounds standardized using urine specific gravity is higher than that normalized using creatinine standardization.

**Figure 1:**
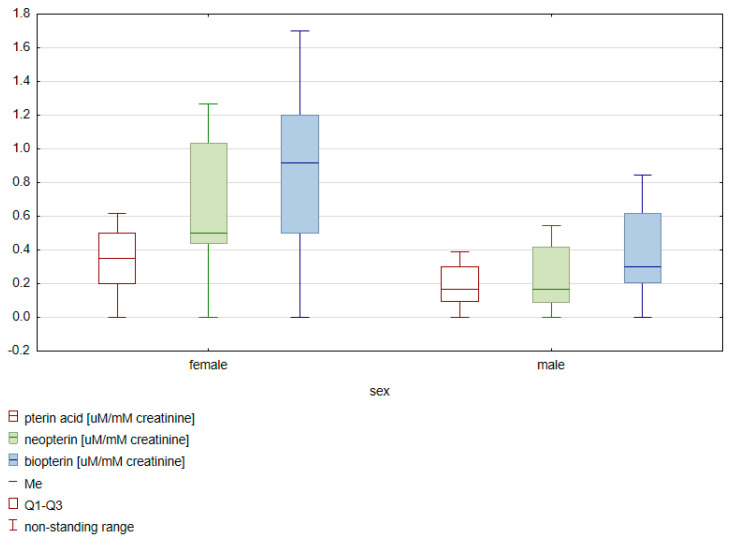
Illustration of a statistically significant difference in the control group with regard to sex for creatinine-standardized pterin compounds (pterin acid *p* = 0.038, neopterin *p* = 0.019, biopterin *p* = 0.044).

An analysis was carried out to determine possible differences between the sexes for the obtained concentration values of pterin compounds. A comparative analysis was performed in patients from the control group and patients suffering from bladder cancer. In the group of bladder cancer patients, no statistically significant differences were found in the concentrations of pterin compounds between men and women, taking into account both creatinine normalization and specific gravity. On the other hand, in the control group, significantly higher concentrations of pterin acid (*p* = 0.038), neopterin (*p* = 0.019) and biopterin (*p* = 0.044) were observed in women when the values were standardized for creatinine (Figure 1).

**Figure 2:**
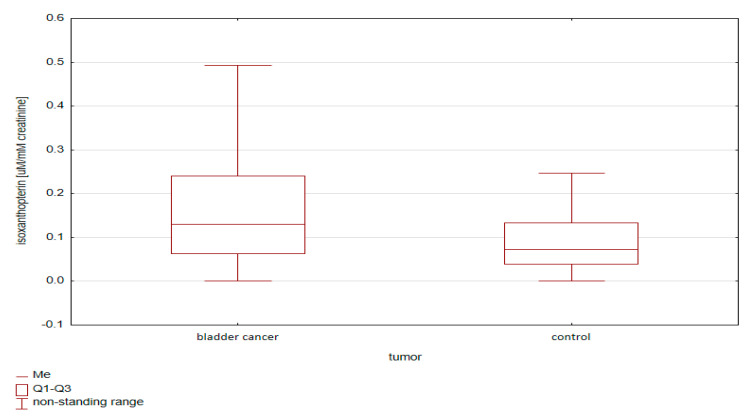
Comparison of isoxanthopterin concentrations standardized to creatinine between bladder cancer patients and controls, regardless of gender.

**Figure 3:**
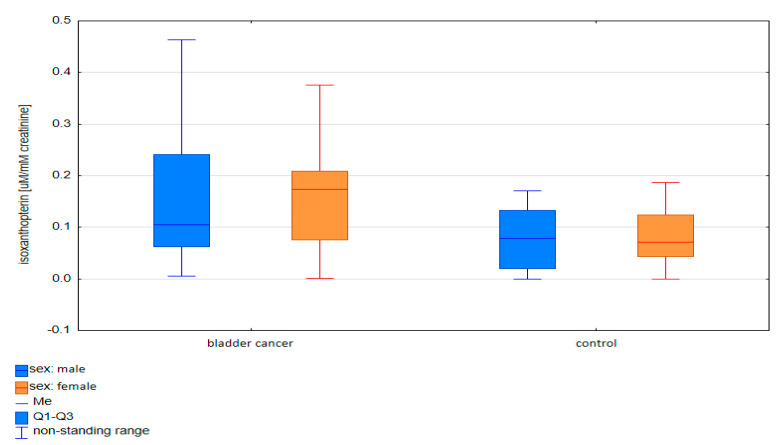
Comparison of isoxanthopterin concentrations standardized to creatinine between bladder cancer patients and the control group in the male and female groups.

The differences in the pterin concentrations between the groups (bladder cancer and control group) were then compared, taking into account various standardization methods. Both when analyzing the group of bladder cancer patients in total (Figure 2) and taking into account the division into sex (Figure 3), a statistically higher concentration of isoxanthopterin was observed in the group of cancer patients compared to that in the control group (*p* < 0.05).

More statistically significant differences were observed for standardization to urine specific gravity. Distinctions in the levels of isoxanthopterin, xanthopterin, neopterin and pterin acid were observed between various groups of patients studied (Table 1).

#### 2.1.3. Cluster Analysis

A dendrogram prepared by Ward’s method was used to agglomerate the variables and assess their similarity. Euclidean distance was used as a measure of distance for agglomeration analysis [40].

**Figure 4:**
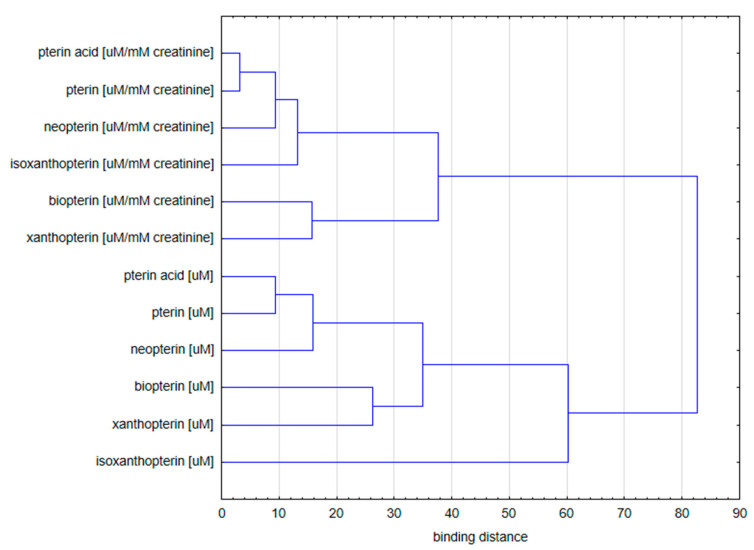
Dendrogram of variables used for analysis in group of bladder cancer patients and control group.

It is noteworthy that the agglomeration of the variables in the bladder cancer patients and the controls group shows two separate clusters (Figure 4):

1. Concentration of pterin compounds with standardization to creatinine;
2. Concentration of pterin compounds with standardization to urine specific gravity.

In the second cluster, two sub-clusters can be distinguished:

(a) Isoxanthopterin concentration;
(b) Other pterin compounds standardized to specific urine gravity.

Hierarchical analysis shows that the concentration of isoxanthopterin differed significantly from all the other values of the parameters tested.

### 2.2. Multivariate Analysis

Multidimensional methods enable the analysis of the structure of connections and interdependencies between many variables. This type of analysis usually comes down to reducing or simplifying the data structure, classifying variables or objects into specific groups, identifying interdependencies between variables, predicting relationships between them, and constructing and testing hypotheses [41].

#### 2.2.1. Principal Component Analysis (PCA)

The main applications of factor analysis techniques, including principal component analysis, are (1) the reduction of the number of variables and (2) structure detection in relationships between variables. PCA is based on transforming the original variables into a number of new, uncorrelated variables called principal components. Each principal component (PC) is a linear combination of the original variables [42].

**Figure 5:**
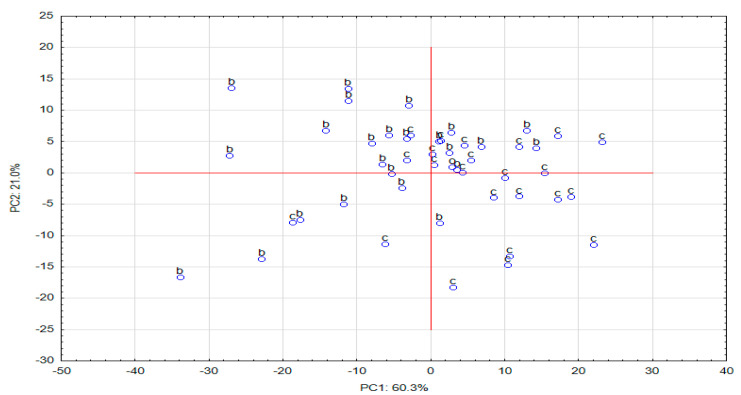
Absolute principal component scores of cases based on covariance for aggregated data (b—bladder cancer, c—control).

In order to determine the factors that could group patients suffering from bladder cancer and patients from the control group, the main component analysis was performed in three stages. In the first stage, data standardized to creatinine were used; in the second, those standardized to specific gravity; and in the third, aggregated data. In order to determine the factors that could group patients suffering from bladder cancer and patients from the control group, the main component analysis was performed in three stages. In the first stage, data standardized to creatinine were used; in the second, those standardized to specific gravity; and in the third, aggregated data. The second, third and fourth principal components, explaining 98.9%, 94.4% and 95.3% of the original variability, respectively, were selected. The results of the analysis do not allow the observation of clear clusters of cases (Figure 5).

#### 2.2.2. Classification Analysis (Decision Trees)

Later in the analysis, the construction of a classification model was started, which will classify patients as healthy or suffering from cancer of the urinary tract with the highest efficiency. Classification trees are used to build predictive models (predicting subsequent data based on the model based on previously provided information) and descriptive models. A tree is a graphical model derived from dividing a set of observations into disjoint subsets in order to obtain subsets that are as homogeneous as possible from the dependent-variable-value point of view. Building a tree is a multistage process where all predictors are analyzed at each stage and the one providing the most homogenous subsets is selected [43].

The general models of regression and classification trees were used to build the classification model. The aim of analysis, among other things, was to find out which method of standardizing the concentrations of pterin compounds would allow the better classification of patients.

**Figure 6:**
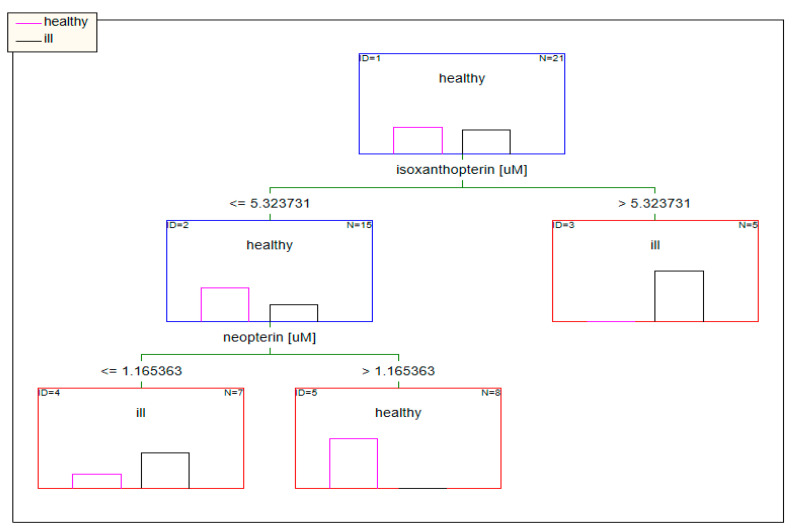
Classification model based on data standardized to urine specific gravity built using decision trees.

When the concentrations of pterins standardized to urine specific gravity were used as input data, factors such as the concentration of isoxanthopterin and neopterin in the tree model determined the classification of patients (Figure 6).

**Figure 7:**
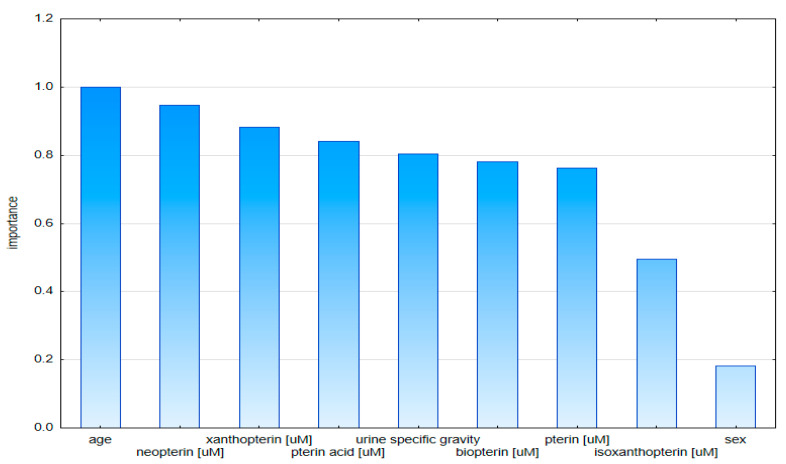
Graph of validity of model variables based on urine specific gravity-standardized data.

Nevertheless, the plot of the importance of the model variables based on the data standardized to urine specific gravity indicates the age and the concentration of neopterin and xanthopterin as having the greatest impact in the classification of patients in the first place (Figure 7).

**Figure 8:**
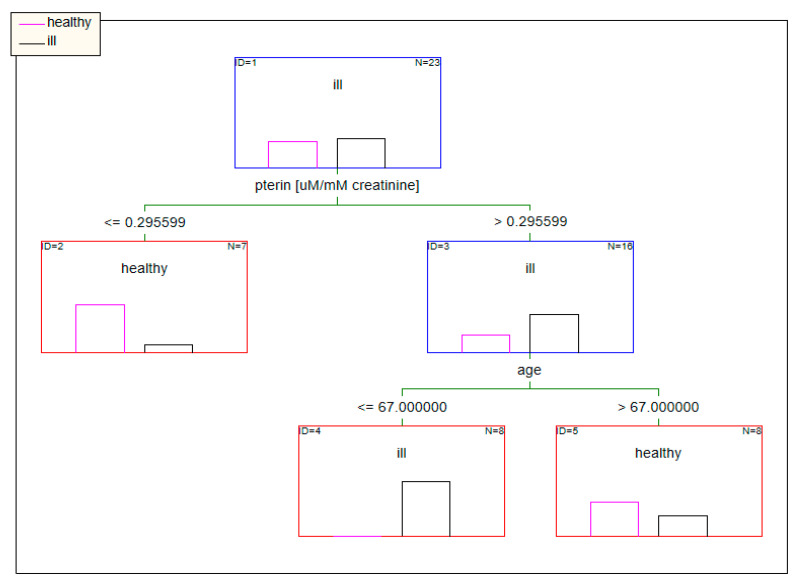
Classification model based on data standardized to creatinine built using decision trees.

When the concentrations of pterins standardized to urine creatinine were used as input data, factors such as the concentration of pterin and age in the tree model determined the classification of patients (Figure 8).

**Figure 9:**
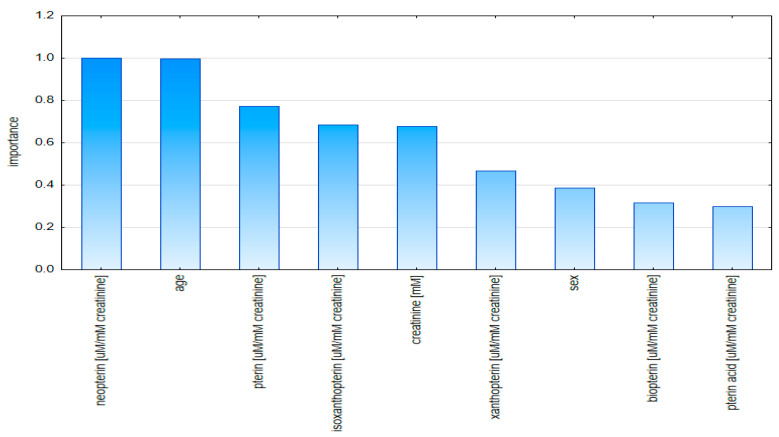
Graph of validity of model variables based on creatinine-standardized data.

The plot of the importance of the model variables based on the data standardized to creatinine indicates, in first place, the concentration of neopterin and age as having the greatest influence in the classification of patients (Figure 9).

**Figure 10:**
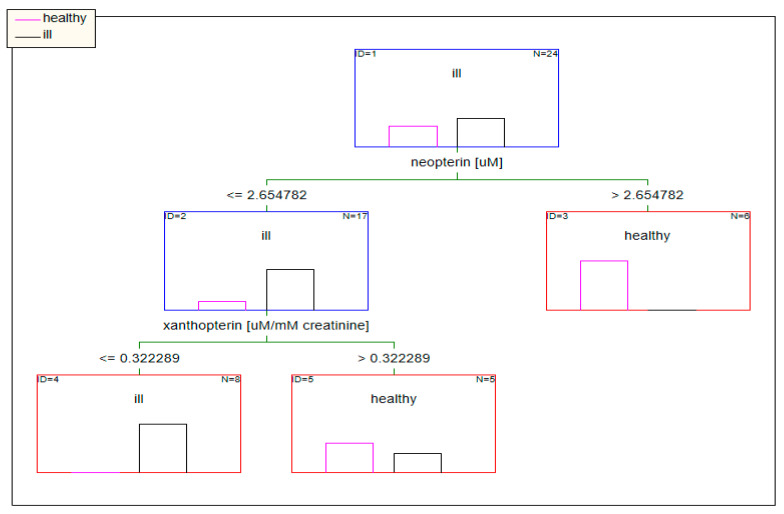
Classification model based on aggregated data built using decision trees.

Using all the data (pterin compounds standardized to creatinine and urine specific gravity) to build classification trees, the final classification factors were the concentration of neopterin standardized to urine specific gravity and xanthopterin converted to creatinine (Figure 10).

**Figure 11:**
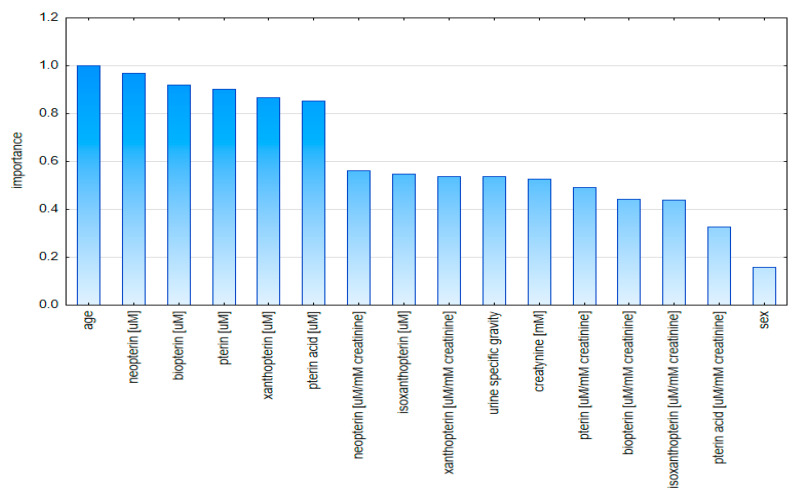
Graph of validity of model variables based on aggregated data.

The plot of the importance of the model-variable-based aggregated data indicates the age and the concentrations of neopterin and biopterin standardized to specific gravity as being the most important (Figure 11).

The use of decision trees allowed the obtainment of three classification models to assess whether a given patient may suffer from bladder cancer based on experimental data standardized according to the two most popular methods and using aggregated data. The sensitivity (SEN), specificity (SPE), positive predictive value (PPV), negative predictive value (NPV), positive reliability ratio (LR_PLUS_), negative reliability ratio (LR_MINUS_) and accuracy (Acc) were used to evaluate the model.

The model based on aggregated data was characterized by high values of SEN and the highest SPE, Acc and LR_PLUS_ (Table 2).

The model based on data standardized to creatinine has a higher SEN and lower SPE than the model based on data standardized to creatinine and aggregated data. An important observation is also that the model based on aggregated data has a higher value of PPV and LR_PLUS_. In the light of these data, it can be assumed that the use of aggregated data (standardized to the specific gravity of urine and creatinine) allows for the better classification of patients as healthy or suffering from bladder cancer.

**Figure 12:**
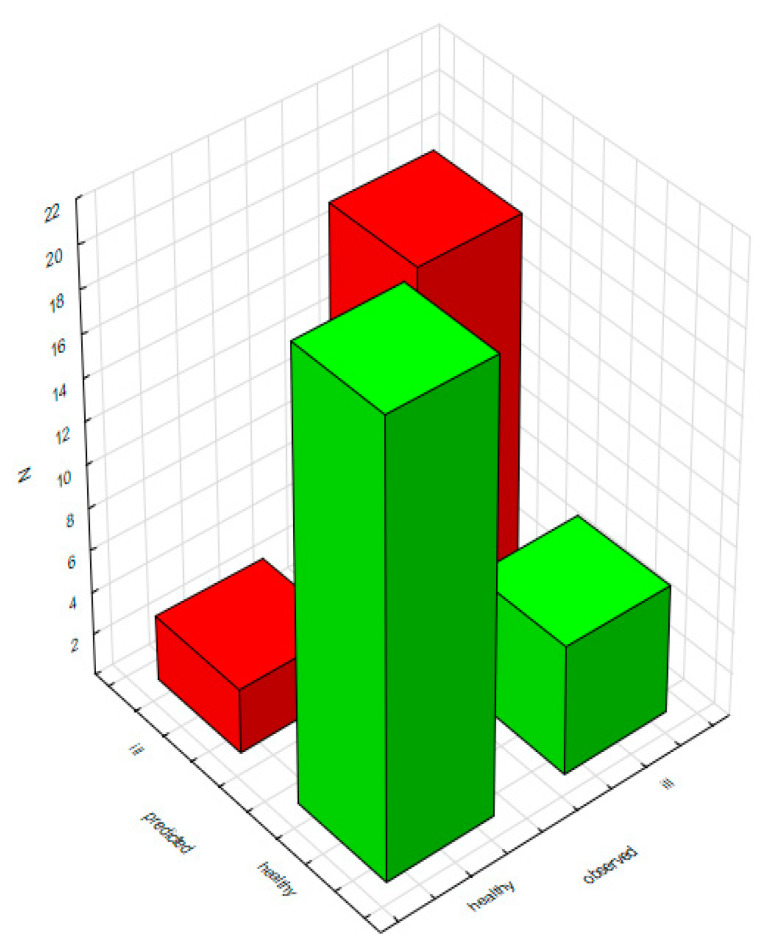
Matrix of patient classification made by a model based on aggregated data.

Therefore, in order to identify patients with bladder cancer with an accuracy of 81.3%, it is necessary to measure the concentrations of neopterin, xanthopterin and creatinine in urine and determine the specific gravity of urine. Importantly, the use of a model based on the above parameters allows a six times greater chance of the correct diagnosis of a person suffering from bladder cancer than of the diagnosis of the same cancer in a healthy person (Figure 12).

The application and development of this model may be an effective method for the non-invasive detection of bladder cancer.

## 3. Materials and Methods

### 3.1. Chemicals and Materials

Deionized water, purified with the Direct-QUV (Millipore, France) system, was used for all aqueous solutions. Acetonitrile and methanol of HPLC grade were purchased from Chempur (Piekary Śląskie, Poland). Phosphoric acid was purchased from Sigma-Aldrich (Switzerland); sodium hydroxide, from P.P.H. Stanlab (Lublin, Poland). Disodium hydrogen phosphate and monosodium phosphate were purchased from POCH S.A (Gliwice, Poland).

The pterin standards L-biopterin, D-neopterin, pterin, pterin 6-carboxylic acid, isoxanthopterin and xanthopterin were purchased from Dr B. Schircks Laboratories (Jona, Switzerland).

### 3.2. Samples and Specimens

There were 31 patients diagnosed with bladder cancer. The control group consisted of 32 healthy people. All participants provided written consent, and the collection was approved by the bioethics commission. The first morning urine sample was used for examination. Collected urine samples were frozen at −80 °C until analysis.

### 3.3. Assays

Prior to analysis, urine samples were thawed at room temperature and oxidized with I2/I− in an alkaline medium. The solution was finally diluted with 10 mM phosphate buffer, pH 7, to a final volume. The sample was filtered through a nylon filter before injection. The chromatographic analysis was carried out using the method proposed by Kośliński et al. [44]. LC analysis was performed by the means of Shimadzu HPLC equipment with a fluorescence detector. The chromatographic system consisted of a C_8_ LiChrospher 60 RP-Select B 250 mm × 4.0 mm, 5 μm chromatographic column, fluorescence detector with ex/em 280/444 wavelengths. This method enabled the separation of six pterin compounds (pterin acid, neopterin, xanthopterin, isoxanthopterin, biopterin and pterin). The determined concentrations of the six pterin compounds were further normalized against creatinine and USG.

### 3.4. Statistical Analysis

The statistical analysis was performed using Statistica 13.1 (StatSoft, Tulsa, OK, USA) and Excel 2010 (Microsoft, Redmond, WA, USA).

The type of distribution was examined using the Shapiro–Wilk test. The Mann–Whitney U test was used to assess differences between groups. In the event of statistically significant differences, the data were visualized using a box plot. For the agglomeration of variables and assessment of their similarity, a dendrogram was prepared using the Ward method. Euclidean distance was used as a measure of distance for agglomeration analysis. Principal component analysis was used to classify correlation coefficients. The correlation matrix was used as an input. To isolate the number of principal components, the Cattell and Kaiser criteria were used.

The sensitivity (SEN), specificity (SPE), positive predictive value (PPV), negative predictive value (NPV), positive reliability ratio (LR_PLUS_), negative reliability ratio (LR_MINUS_) and accuracy (Acc) were used to evaluate the model.

The general models of regression and classification trees were used to build the classification model. The collected data were divided in a 4:1 ratio into learning and testing groups. All analyses were performed at a significance level of 5% (α = 0.05).

## 4. Conclusions

The main objective of the presented study was to evaluate the usefulness of pterin compounds in the diagnosis of bladder cancer, with particular emphasis on the role of creatinine and urine specific gravity as factors for normalizing the concentration of pterin compounds in urine.

The standardization of the concentration of pterin compounds to urine specific gravity allows the building of better classification models for screening patients with potential cancer of the bladder. Overall, for the commonly used methods for standardizing the concentration of pterin compounds in urine, it can be said that the choice of a specific standardization method for these substances in biological material significantly affects statistical inference. The observations of non-identical differences between the studied groups can lead to different conclusions.

Among the compounds that make up the pterin profile, isoxanthopterin can potentially be described as a biomarker of urinary tract cancers; however, the use of aggregated data (standardized for specific gravity of urine and creatinine) allows the better classification of patients as healthy or suffering from bladder cancer.

The obtained results should be approached with great caution, and the diagnostic value of the obtained results requires further confirmation. Although the results do not prejudge the applicability of pterin compounds in the diagnosis of bladder cancer, these results may have an impact on the study of bladder cancer biomarkers and the assessment of the diagnostic value of pterin compounds. Further studies on larger samples of both bladder cancer patients and control groups should be performed to assess the potential diagnostic value of pterins in the context of bladder cancer.

## Figures and Tables

**Table 1:** Significant differences between cancer group and control for variables standardized to urine specific gravity; comparisons (*p*-value).

Compounds	Women *p*-Value	Men *p*-Value
Isoxanthopterin	0.018	0.562
Xanthopterin	0.066	0.003
Neopterin	0.030	0.117
Pterin acid	0.043	0.001
Pterin	0.038	0.030

**Table 2:** Comparison of classification parameters of a model based on a decision tree.

	Standardization for Urine Specific Gravity	Standardization for Creatinine	Aggregated Data
SEN	0.806	0.833	0.750
SPE	0.808	0.480	0.875
PPV	0.833	0.606	0.857
NPV	0.778	0.750	0.778
LR_PLUS_	4.194	1.603	6.000
LR_MINUS_	0.240	0.347	0.286
Acc	0.807	0.653	0.813
